# Genome sequences of three plastic-debris-inhabiting fungi isolated from New Zealand waste and recycling management plants

**DOI:** 10.1128/mra.00266-24

**Published:** 2024-08-01

**Authors:** Nikola Palevich, Shuyan Wu, Yang Li, Faith P. Palevich, Paul H. Maclean, Jaspreet Singh Sidhu, Pornchanok Subharat, Ruy Jauregui, Jana L. Müller, Kerri Reilly, John Mills, Graeme T. Attwood, Gale Brightwell

**Affiliations:** 1 AgResearch Ltd., Grasslands Research Centre, Palmerston North, New Zealand; 2 AgResearch Ltd., Hopkirk Research Institute, Palmerston North, New Zealand; 3 Institute of Agricultural Sciences, ETH Zurich (Eidgenössische Technische Hochschule Zürich), Zurich, Switzerland; 4 Animal Health Laboratory, Biosecurity New Zealand, Ministry for Primary Industries, Wallaceville, New Zealand; 5 Kantonale Veterinäramt Zürich (Zürich Cantonal Veterinary Office), Zürich, Switzerland; University of Maryland School of Medicine, Baltimore, Maryland, USA

**Keywords:** *Cladosporium*, *Epicoccum*, fungi, plastic waste, genome

## Abstract

*Cladosporium* and *Epicoccum* are cosmopolitan fungi of the class Dothideomycetes with few cultured and genomic representatives. Here, we report draft reference genome sequences of *Epicoccum* sp. F181 (GenBank accession number JAJSLS01), *Cladosporium* sp. F165 (JAJSLR01), and F190 (JAJSLT01) isolated from recycling and waste management facilities in New Zealand.

## ANNOUNCEMENT

Members belonging to the Dothideomycetes classes of ascomycetes are diverse, with biofouling and degradative abilities of synthetic plastic polymer materials (1–4). In 2018, *Epicoccum* sp. F181 and *Cladosporium* sp. F165 were originally isolated from recycled plastic trays collected from the Awapuni Resource Recovery Park (Palmerston North, New Zealand), whereas *Cladosporium* sp. F190 was isolated from the Marton Landfill (Marton, New Zealand). Fungi were cultured for 5 days into 10 mL minimal salt medium (MSM) at 25°C (5) supplemented with glucose and low-density polyethylene (LDPE). The presented draft reference genomes are valuable resources for future studies investigating genome evolution of potentially novel fungal species able to colonize plastic surfaces.

For genomic sequencing of each isolate, cryostocks were revived and mycelium was grown for 5 days in 80 mL potato dextrose broth (Sigma-Aldrich) at 25°C and washed with phosphate-buffered saline at 5,000 × g for 5 min. Genomic DNA was extracted by grinding in liquid nitrogen and extracting with Zymo Fungal/Bacteria DNA Miniprep Kit (Zymo Research) and was purified using Zymo Clean & Concentrator kit (Zymo Research), following the manufacturer's instructions. DNA quantity, quality, and integrity were assessed using the High Sensitivity DNA LabChip Kit on the Bioanalyzer 2100 (Agilent Technologies). Each fungal strain was identified by PCR amplification of the Internal Transcribed Spacer (ITS) region using ITS4 and ITS5 primers and amplification protocol (6), sequenced on an ABI 3730 DNA system (Applied Biosystems), and alignment to the NCBI refseq ITS database using BLAST v2.9.0 (7). Strain identities were confirmed (99% identity) for *Epicoccum* sp. F181 (GenBank accession number PP896550) as *Epicoccum endophyticum* (GenBank accession number NR_172436), *Cladosporium* sp. F165 (GenBank accession number PP896549) as *Cladosporium chasmanthicola* (NR_152307), and *Cladosporium* sp. F190 (GenBank accession number PP896551) as *Cladosporium pseudocladosporioides* (NR_152296).

For each isolate, approximately 12 µg of genomic DNA was sequenced using a combination of Illumina and PacBio Single Molecule Real Time (SMRT) technologies (Table 1). For Illumina, 250 bp paired-end reads were sequenced using the Nextera XT DNA library prep kit v2 (Illumina) on the MiSeq platform. The SolexaQA++ software v3.1.7.3 (8) was used to check the quality of the raw reads and perform read trimming with standard parameters. Prior to library preparation for PacBio sequencing, Covaris g-TUBEs (Covaris) were used to fragment the genomic DNA, AMPure PacBio beads (PacBio) for purification, and BluePippin system (Sage Science) to select for fragment size of >20 kb. DNA fragments were end-repaired to construct SMRTbell DNA template libraries with a Bioanalyzer used to assess quality and fragment size estimation. Long-read libraries were prepared using the SMRTbell DNA CLR template prep kit v2.0 (PacBio) and sequenced on three SMRT cells using a Sequel II v8.0 system in continuous long read (CLR) mode with SMRT Link v11.1 used for data acquisition. Reads were preprocessed using Fastp v0.21.0 with a > Q30 threshold (9), followed by a hybrid *de novo* assembly correcting the refined PacBio reads with Illumina trimmed data performed with Canu v2.2 (10). We polished the assembly with one round of PILON v1.24 (11). Assembly quality and completeness were evaluated by BUSCO v5.4.4 (12) using the lineage “fungi_odb10 database”. Default parameters were used for all software unless otherwise specified. The sequencing data metrics and genome features are summarized in Table 1.

**TABLE 1:** Genomic features and assembly statistics of representative *Epicoccum* sp. and *Cladosporium* sp. genomes

Metadata			
Assigned taxonomy	*Epicoccum* sp.	*Cladosporium* sp.	
Strain identifier	F181	F165	F190
Isolate name alias	Lan181-CD2	Lan165-M2b	Rec190-CD2b
NCBI Taxonomy ID	2904842	2904843	2904841
Isolation source	Marton Landfill		Awapuni Resource Recovery Park
Isolation Country	New Zealand		
Genome project information			
GenBank accession numbers	JAJSLS01	JAJSLR01	JAJSLT01
BioSample ID	SAMN24012772	SAMN24012771	SAMN24012773
Assembly ID (GCA)	GCA_037042865	GCA_037042895	GCA_037042825
PacBio data			
SRA accession|Run IDs	SRX15217855|SRR19151143	SRX15217856|SRR19151142	SRX15217854|SRR19151144
Total read length (bp)	4,683,302,991	4,052,731,060	4,645,967,316
No. of subreads	353,079	292,837	352,112
*N*_50_ read length (bp)	17,586	17,706	17,460
Illumina data			
SRA accession|Run IDs	SRX13451535|SRR17273850	SRX13451534|SRR17273851	SRX13451536| SRR17273849
Total read length (bp)	1,657,148,644	1,928,529,818	1,925,560,242
No. of filtered reads (bp)	1,356,558,704	1,651,738,291	1,562,163,222
Genome assembly statistics			
Assembly size (bp)	36,126,079	34,560,626	33,867,326
Genome coverage (×)	130	117	137
G + C content (%)	51.6	52.5	52.6
DNA contigs	40	40	34
Contig *N*_50_ (bp)	1,412,226	2,068,171	1,945,840
Contig L50	9	8	8
BUSCO C| M (%)^a^	99.3|0.7	99.7|0.3	99.6|0.4

^a^ C and M refer to complete and missing BUSCO fungal genes (*n* = 758).

## Data Availability

The whole-genome sequence data and raw sequences are available at NCBI under the BioProject accession number PRJNA788622. Detailed information regarding GenBank, BioSample, Assembly, and Sequence Read Archive (SRA) accession number identifiers can be found in Table 1.
